# Analysis of Dexmedetomidine on the Quality of Awakening During Neurosurgery

**DOI:** 10.1515/tnsci-2019-0026

**Published:** 2019-07-12

**Authors:** Jing Cao, Hui Li, Shengwen Song, Xuyan Zhou, Xu Shen

**Affiliations:** 1 Medical Center for Anesthesia and Pain, First Affiliated Hospital of Jiaxing College, Jiaxing, China; 2 Department of Anesthesiology, First Affiliated Hospital of Medical College of Zhejiang University, Hangzhou, China

**Keywords:** dexmedetomidine, neurosurgery, wake-up, data fusion algorithm

## Abstract

Dexmedetomidine has a dose-dependent sedative and analgesic effect. To further evaluate the wake-up quality of dexmedetomidine in patients undergoing neurosurgery, a meta-analysis of dexmedetomidine in a randomized controlled trial of general anesthesia was performed. Firstly, an experimental algorithm was proposed, and then the data fusion algorithm was used to conduct randomized controlled trials. The clinical efficacy and safety of dexmedetomidine in the acupuncture of neurosurgical patients were evaluated one by one for quality evaluation and data extraction. The effect of different input variables on the depth of anesthesia was studied by using a multi-data fusion approach. The results show that the data fusion algorithm proposed can effectively connect redundant information and complementary information in multiple data, and estimate the real parameters of the measured object. In addition, data fusion brings great convenience to the design of control algorithms and controllers, and provides an effective basis for system simplification. Experiments have shown that dexmedetomidine is effective and safe in the operation of neurosurgical motor function, and the management of the recovery period is safe and effective. Based on the research, it can provide some reference for the awakening of patients undergoing neurosurgery, and promote the progress and development of medicine.

## 1 Introduction

A variety of new monitoring methods have been widely used in clinic with the rapid development of medicine, such as motor evoked potentials and functional magnetic resonance. However, these methods also have their limitations, such as brain shift caused by space-occupying lesions, signal interference, false positive rate, individual differences, etc [1]. Studies have shown that awakening patients during craniotomy is one of the effective ways for patients to avoid impaired important functions in functional areas. Neurosurgeons are still the first choice for craniotomy [2]. In functional neurosurgery, in order to improve the benefits of traumatic resection and reduce the risk of neurological dysfunction, patients often require neurophysiological tests during surgery to determine changes in the surgical site or expected function, which requires an anesthetic that should be sedated and analgesic [3]. Dexmedetomidine (Dex) is a special adrenergic receptor agonist with sedative, analgesic, antisympathetic, mild respiratory depression, and easy to wake up. These features offer the possibility of intraoperative anesthesia selection [4]. In this context, a study of the effect of dexmedetomidine on the quality of intraoperative wake-up in patients undergoing neurosurgery was developed.

Dexmedetomidine sets wake-up quality in patients undergoing neurosurgery were studied. Firstly, an experimental algorithm was proposed, and then a data fusion algorithm was used to conduct a randomized controlled trial to observe the clinical efficacy and safety of dexmedetomidine in the arousal quality of patients undergoing neurosurgery. Quality evaluation and data extraction were performed one by one. The method of data fusion studied the effects of different input variables on the depth of anesthesia. It effectively contacts the redundancy and complementary information in the multi-data, and estimates the real parameters of the measured object as much as possible.

The effect of dexmedetomidine on the quality of intraoperative wake-up in patients undergoing neurosurgery was investigated. Firstly, the status quo of the research on the wake-up quality of dexmedetomidine and neurosurgery patients was expounded, in order to provide the theoretical basis for the following. Then the principle of quantitative inhalation anesthesia was analyzed, and the data fusion algorithm was proposed. The characteristics, main principles and implementation flow of the algorithm are analyzed. Finally, the data fusion algorithm was tested, and the data was normalized, and the four signals of blood pressure, muscle relaxation, CO2 concentration and pulse were processed, and the experimental conclusion was obtained.

## 2 Related Work

In 1999, the US Food and Drug Administration approved the initial sedation of patients with mechanical ventilation in the 24-hour intensive care unit, and China has only begun to use dexmedetropine in recent years. Luo X et al believe that surgery for brain damage is likely to cause severe postoperative nerve damage. How to accurately locate and remove lesions while ensuring normal neurological function is a problem faced by psychiatrists and mental health doctors [5]. Thongrong C et al. used neuronavigation and electrophysiological techniques to locate neurological anatomical functions during awake state during surgery to avoid damage to the functional area [6]. Suero M E and other studies found that the

anti-sympathetic effect of dexmedetomidine reduced perioperative cardiovascular events and reduced mortality [7]. Mathews L and other studies found that intravenous dexmedetomidine can significantly reduce the perioperative heart rate variability of upper abdominal surgery in elderly patients with coronary heart disease, and inhibit the perioperative surgical stimulation of cardiac autonomic nerve function. It has cardioprotective effects and reduces the incidence of cardiovascular adverse events such as myocardial ischemia and arrhythmias [8]. Studies by Morace R et al have shown that preoperative intramuscular injection of dexmedetomidine is effective in stabilizing perioperative hemodynamic stability while reducing oxygen consumption and reducing high metabolism [9]. Honoratocia M C and other recommendations for intraoperative recovery require higher requirements for anesthesia management. Patients should be removed during lesion localization and resection to avoid interference with anesthetic drugs on neurophysiological monitoring, cerebral cortical neuroanatomy and functional localization, and operator response to assessment of neurological functional status [10]. Mai L G M et al proposed that propofol has rapid recovery and neuroprotection under total intravenous anesthesia, and is often used to awaken patients in brain functional area surgery. The study found that the duration and quality of arousal are often unsatisfactory, and it is easy to cause increased intracranial pressure due to asphyxia and agitation [11].

As a novel, highly selective, adrenergic receptor agonist, dexmedetomidine has a variety of functions including dose-dependent sedation, analgesia, anxiety, inhibition of sympathetic nerves and brain protection. Without respiratory depression, the receptor can excite the brainstem blue spot (awake and sleep), the most densely packed area of the central nervous system, responsible for mediating and maintaining a natural non-sleep state [12]. Similar to physiological sleep, it is easy to wake up and has fewer side effects. It is an ideal drug for awakening in functional neurosurgery. There are few domestic reports and it still needs to accumulate data.

## 3 Information and methods

### 3.1 General Information

With the application and development of intraoperative neuroelectrophysiological monitoring technology in clinical practice, neurosurgery has changed from traditional anatomical mode to modern anatomical mode, and the quality and effect of surgery have been greatly improved.This experiment was performed in patients with scoliosis orthopedic surgery. The patient had 40 cases of 24 male and female patients, 16 cases, Asa~IIIII. the range was basically 16-40 years old, 35 ~ 65 kg, and the body mass of liver and kidney function was Normal; no mental illness, neuromuscular disease or hearing abnormalities. Excluding patients with severe cardiopulmonary dysfunction, 18 patients had mild preoperative pulmonary function and 22 patients had normal lung function. This experiment has been approved by the ethics committee. The patient should be informed of the method and necessity of awakening anesthesia during the first hour before surgery to minimize the psychological burden and to sign an informed consent form.

### 3.2 Anesthesia method

Drinking alcohol was prohibited before surgery. Fasting for 10 hours, local anesthesia was performed by radial artery puncture into the operating room, conventional ECG monitoring, and induction of central venous puncture. Preoperative medication: long toning 0.1mg/ kg, induced drugs: midazolam 0.05mg/kg, sufentanil 0.4μg/kg, propofol induced dose 3~4μg/mL, rocuronium 0.6mg /kg, maintained with propofol 3~8μg/mL and remifentanil 0.1~0.2μg/(kg·h) during the operation, using rocuronium bromide. The BIS value during anesthesia was kept at 40~ Between 50, the end-tidal CO2 concentration is maintained between 35 and 45 mmHg. After extubation, the anesthesia recovery room was closely observed.

### 3.3 Experimental design

The wake-up quality of dexmedetomidine in neurosurgery patients was studied.Firstly, an experimental algorithm is proposed, and then a randomized controlled trial is conducted using data fusion algorithm to observe the clinical efficacy and safety of dexmedetomidine in the wake-up quality of Neurosurgery patients. The quality evaluation and data extraction are carried out one by one.In this paper, the effect of different input variables on anesthesia depth was studied by means of multi-data fusion. Effectively link redundant and complementary information in multi-data to estimate the real parameters of the tested object as much as possible.In this study, 40 patients were randomly divided into two groups (n=20): before anesthesia induction, group D was pumped with 1 μg/kg dexmedetomidine for 30 min, and the induction intubation was completed at 0.5 μg/(kg·h). Intubation was induced. Group N: Pumping was done in the same manner by replacing with the same amount of physiological saline. Group N: Replacing the same amount of normal saline and complete the pumping in the same manner. 45 minutes before the start of the experiment, the dose of dexmedetomidine and physiological saline pump was reduced to 0.2 μg/(kg·h), and the drug was stopped for 15 minutes before waking up. The pump was paused 45 minutes before the operation to allow the patient to wake up and extubate. Careful recording of wake-ups is required to wake up at respiratory recovery time (T1), wake-up time (T2), and 15 minutes before the end, wake up immediately, and wake up with a heart rate (HR), mean arterial pressure (MAP), and BIS. When you are a teacher’s Ramsey calm score, 1 day to ask the patient to wake up after the time of the vascular score and patient satisfaction.

### 3.4 Wake-up test

During the wake-up period, stop using other drugs, the agile and saline syringe pump is 0.2μg / (kg · h) to stay awake until the end. On both sides of the spine, artificial respiration should be performed every 30 minutes. After the patient is awake, the patient is fully awakened and the anesthesia continues to be strengthened after the test. After the operation, the wake-up quality grading standard was evaluated with reference to [6]. It is mainly divided into four grades, level 1: the patient is awake after hearing the sound, and can respond accordingly according to the requirements; level 2: the patient can barely open his eyes after hearing the sound, and can respond to the request according to the requirements; level 3 : After hearing the sound, the patient blinked violently, unable to respond according to the requirements, and often had limb movements; Level 4: After hearing the sound, the patient opened his eyes sharply and had a very significant agitation. Not conducive to the stable advancement of surgery.

### 3.5 Statistical processing

The SPSS 17.0 tool was selected for research, and the rank sum test was used to test the wakeup quality and sedation score of the measured data. Other measured data were explained by x±s. The t test and χ2 test were used for the two groups. P<0.05 was considered statistically significant. T-test uses t-distribution theory to infer the probability of difference occurrence, so as to compare whether the difference between the two averages is significant. Chi-square test is a widely used hypothesis test method. It is applied in statistical inference of classification data, including: chi-square test for comparison of two or two constituent ratios, chi-square test for comparison of multiple or multiple constituent ratios, and correlation analysis of classification data.

## 4 Research results

The blood flow dynamics of patients in group D were stable when they were awake, and the blood pressure and heart rate were stable when they were awake. Hemodynamic changes were significant at different time points in group N (P<0.05). The difference between the two groups was statistically significant (P<0.05). There was no significant difference in the BIS values between the two groups during the awakening stage, as shown in Table 1-3. There was no significant difference in respiratory recovery time and recovery time between the two groups (P>0.05), and the difference in recovery quality was statistically significant (P<0.05). The number of agitation in group N was significantly higher than that in group D (P<0.05), as shown in Table 4. There was a statistically significant difference in Ramsay sedation score between the two groups (P<0.05), as shown in Table 5. There was a statistically significant difference between the two groups in VAS score and postoperative satisfaction score (P<0.05), as shown in Table 6.

**Table 1:** Changes of HR in two groups of patients(frequency/min,x±s)

group	10 minutes before anesthesia	15 minutes before awakening	Awaken instantly	Wake up	Extubation time
Group D	78.1±11.2	63.2±10.1	76.5±11.7	77.7±12.0	69.4±10.6
Group N	80.1+10.2	66.8±10.1	85.3±11.6	98.1±11.7	85.6±11.1
T value	0.53	1.1	0.65	5.41	4.71
P value	>0.05	>0.05	>0.05	<0.05	<0.05

**Table 2:** Changes of MAP in two groups of patients( mmHg,x± s)

group	n	10 minutes before anesthesia	15 minutes before awakening	Awaken instantly	Wake up	Extubation time
Group D	20	101.3±11.1	71.7±7.2	72.8±8.1	75.8±7.7	71.6±7.2
Group N	20	98.5±11.2	78.3±7.2	88.2±6.4	101.2±7.4	88.5±8.2
T value		0.37	1.08	1.86	8.35	7.02
P value		>0.05	>0.05	<0.05	<0.05	<0.05

**Table 3:** Changes of BIS values in two groups of patients( x± s)

group	n	10 minutes before anesthesia	15 minutes before awakening	Awaken instantly	Wake up	Extubation time
Group D	20	95±2.4	66±2.7	84±3.7	88±2.9	91±2.3
Group N	20	92±3.2	67±2.8	83±3.7	87±3.6	92±2.9
T value		1.07	3.32	0.86	0.77	1.20
P value		>0.05	<0.05	>0.05	>0.05	>0.05

**Table 4:** Comparison of breathing recovery time T1, recovery time T2 and awakening quality between two groups( min,x± s)

group	n	T_1_	T_2_	Wake-up quality (n)				Arousal agitation (n)	
				
				Level 1	Level 2	Level 3	Level 4	n	%
Group D	20	15.1±1.5	21.2±1.6	18	0	0	1	0	4%
Group N	20	12.4±1.2	18.3±1.3	6	5	7	1	5	29%
U/χ^2^ value		12.6	1.64		8.14			4.319	
P value		>0.05	>0.05		<0.025			<0.001	

**Table 5:** Ramsay sedation score in two groups

group	n	1point	2point	3point	4point	5point	6point
Group D	20	0	18	1	0	0	0
Group N	20	6	12	1	1	1	0
U value					10.82		
P value					<0.005		

**Table 6:** VAS score and postoperative satisfaction score of patients in two groups

group	n	VAS score						Postoperative satisfaction		
		
		0-2 point	3-5 point		6-8 point			1 point	2 point	3 point
Group D	20	14	5			0	0	0	15	4
Group N	20	7	5			3	2	6	14	1
U value				10.76					10.88	
P value				<0.05					<0.05	

As a newest α2 adenosine receptor agonist, DEX has the characteristics of sedation, analgesia, anxiolytic, and sympathetic inhibition. It is characterized by the ability to reduce the amount of general anesthetic drugs during surgery, and to awaken the cooperative, long-term sedation and no adverse effects of respiration after surgery and surgery. At the same time, it also has the effect of sympathetic inhibition, reducing the loss of armor When adrenaline is released, which helps to reduce the adverse effects caused by stress. Because DEX selectively acts on the alpha 2 adrenergic receptor, it forms a state similar to normal sleep. Unlike other drugs that work on the cerebral cortex, it has a very serious effect on awakening during spinal orthopedic surgery. Ard et al [13] proposed that DEX should be selected in children’s neurosurgical awakening anesthesia, and DEX 0.1~0.3μg/(kg·h) should be injected in this experiment. The dexmedetomidine should be used for waking in the operation. The dose of DEX should be injected into 0.2. Gg/(kg·h).

It can be seen from the results that the hemodynamics of patients in group D before and after waking were more stable than those in group N, which may be closely related to the sympathetic inhibition of dexmedetomidine. Although the patient was able to wake up with agile D-group and low-dose injections at a constant rate, there was no significant difference in respiratory recovery time between the two groups, and there was no statistically significant difference. At the same time, it was also shown that the 0.2μg/(kg·h) pump injection agile calm did not cause the reaction of patients with respiratory depression, and it also demonstrated the Ard scholar study [14]. Awakening both groups of patients during the arousal quality grading period compared to nearly 90% of patients in group D were able to respond to the desired exercise after hearing the sound waking up. According to Ramsay’s sedation score analysis, patients in group D were relatively calm and able to cooperate with surgery [15]. Anxiety and restlessness occurred in 6 of the N patients, indicating that pumping DEX during the waking period allowed the patient to work quietly and keep the patient in a good recovery state. The VAS scores of patients in group D were also significantly lower than those in group N. Most of them were painless or mildly painful. In group N, the pain score was more than 6 points and reached 6 cases. After surgery, in the two groups of patients, in terms of satisfaction, group D and height were relative to group N. There was no memory between the two groups before and after the anesthesia waking up. But the patient can also wake up in memory and wake up to know that he is awakened.

In the experiment, the BIS value of the awakening period is similar to that of TaeKyoung Seol, which is higher than the BIS value obtained by Heleen J. It may be because Heeleen J selected the subjects mainly for adolescents, and DEX in the anesthesia process of compound propofol and remifentanil. The amount of anesthetic drug used may be closely related to the calming and analgesic effects of DEX. Both groups of patients were removed from the tube and sent to the recovery room. In the rehabilitation room, patients in group D had better sedation results. Based on the same analgesic formula, the pain in group D was less than that in group N. The single injection of sufentanil in the anesthesia recovery room was more than the group N, and there were 7 patients with eschar conditions, indicating that DEX was used during surgery. Auxiliary anesthesia helps to avoid postoperative agitation. According to the results of this experiment, it is proposed that pumping DEX 0.2 μg/(kg·h) when the anesthesia of the scoliosis surgery does not cause respiratory depression and wake delay, but Significantly improve the quality of recovery and contribute to the implementation of anesthesia. Intraoperative selection of dexmedetomidine helps reduce the amount of intraoperative sedatives, and helps patients with postoperative pain and avoid postoperative anxiety.

To sum up, dexmedetomidine is a new and highly selective adrenergic receptor agonist, which has many effects such as dose-dependent sedation, analgesia, anti-anxiety, inhibition of sympathetic nerve and brain protection. Dexmedetomidine has no respiratory depression. It can activate the locus coeruleus of the brain stem (responsible for mediating awakening and sleep), which is the most dense receptor of the central nervous system. It can trigger and maintain natural inactive sleep (state, similar to physiological sleep, easy to wake up, with few side effects. It is an ideal drug for awakening in functional neurosurgery.

## 5 Conclusion

With the application and development of intraoperative neuroelectrophysiology and other surgical monitoring techniques in clinical practice, neurosurgery has changed from the traditional anatomical model to the modern anatomical model. The quality and effect of surgery have been greatly improved. Based on the development of interdisciplinary research in biomedical and control theory, the effect of dexmedetomidine on the quality of in-operative wake-up in patients undergoing neurosurgery was analyzed. The platform distributes the big data in different nodes. The Map Reduce algorithm is used in the calculation process for distributed computing, which is equivalent to each node only processing its local data. The results show that the parallelism of data processing is greatly increased, and the system computing rate is increased to reduce network congestion and improve system throughput. Experiments show that the algorithm can reduce computational complexity and improve classification efficiency. The algorithm is suitable for automatic classification of class domains with a larger sample size, especially for the unique “conscious sedation”. Similar to the nature of non-REM sleep, there is no stimulating sleep state. The patient’s speech stimulation is easy to wake up, and cooperates and communicates with the medical staff, stimulates to fall asleep again, and almost does not inhibit breathing. The theory is tested and the effectiveness of the system is demonstrated from both theoretical and practical aspects. The disadvantage of this method is that it is computationally intensive because each text is classified. So it is necessary to calculate its distance to all known samples to get its K nearest neighbors, which still needs further improvement in future research.

## References

[1] Su S, Ren C, Zhang H (2016). The Opioid-Sparing Effect of Perioperative Dexmedetomidine Plus Sufentanil Infusion during Neurosurgery: A Retrospective Study[J]. Frontiers in Pharmacology.

[2] Liu Y, Liang F, Liu X (2017). Dexmedetomidine Reduces Perioperative Opioid Consumption and Postoperative Pain Intensity in Neurosurgery: A Meta-analysis.[J]. J Neurosurg Anesthesiol.

[3] Stambolija V, Miklić B M, Lozić M (2018). Analgosedation With Dexmedetomidine in a Patient With Superior Vena Cava Syndrome in Neurosurgery[J]. J Neurosurg Anesthesiol.

[4] Kwon WK, Kim J H, Lee J H (2016). Microelectrode recording (MER) findings during sleep-awake anesthesia using dexmedetomidine in deep brain stimulation surgery for Parkinson’s disease.[J]. Clinical Neurology & Neurosurgery.

[5] Luo X, Zheng X, Huang H (2016). Protective effects of dexmedetomidine on brain function of glioma patients undergoing craniotomy resection and its underlying mechanism[J]. Clinical Neurology & Neurosurgery.

[6] Thongrong C, Sirikannarat P, Kasemsiri P (2017). Comparison of dexmedetomidine and fentanyl to prevent haemodynamic response to skull pin application in neurosurgery: double blind randomized controlled trial[J]. Anaesthesiol Intensive Ther.

[7] Suero M E, Schipmann S, Mueller I (2018). Conscious sedation with dexmedetomidine compared with asleep-awake-asleep craniotomies in glioma surgery: an analysis of 180 patients[J]. Journal of Neurosurgery.

[8] Mathews L, Camalier CR, Kla KM (2017). The Effects of Dexmedetomidine on Microelectrode Recordings of the Subthalamic Nucleus during Deep Brain Stimulation Surgery: A Retrospective Analysis.[J]. Stereotactic & Functional Neurosurgery.

[9] Morace R, De A M, Aglialoro E (2016). Sedation with α-2 agonist dexmedetomidine during unilateral subthalamic nucleus deep brain stimulation: a preliminary report.[J]. World Neurosurgery.

[10] Honoratocia M C, Martinezsimón A, Guridi J (2017). Sedation during surgery for movement disorders and perioperative neurological complications: an observational study comparing local anesthesia, remifentanil and dexmedetomidine.[J]. World Neurosurgery.

[11] Mai L G M, Ambrus R, Rasmussen R (2017). The effect of dexmedetomidine on cerebral perfusion and oxygenation in healthy piglets with normal and lowered blood pressure anaesthetized with propofol-remifentanil total intravenous anaesthesia[J]. Acta Veterinaria Scandinavica.

[12] Zhang B, Wang G, Liu X (2017). The Opioid-Sparing Effect of Perioperative Dexmedetomidine Combined with Oxycodone Infusion during Open Hepatectomy: A Randomized Controlled Trial:[J]. Frontiers in Pharmacology.

[13] Surve RM, Bansal S, Reddy M (2016). Use of Dexmedetomidine Along With Local Infiltration Versus General Anesthesia for Burr Hole and Evacuation of Chronic Subdural Hematoma (CSDH)[J]. Journal of Neurosurgical Anesthesiology.

[14] Wang W, Lei F, Bai F (2016). The Safety and Efficacy of Dexmedetomidine vs. Sufentanil in Monitored Anesthesia Care during Burr-Hole Surgery for Chronic Subdural Hematoma: A Retrospective Clinical Trial[J]. Frontiers in Pharmacology.

[15] Goettel N, Bharadwaj S, Venkatraghavan L (2016). Dexmedetomidine vs propofol-remifentanil conscious sedation for awake craniotomy: a prospective randomized controlled trial[J]. British Journal of Anaesthesia.

